# Fn3 proteins engineered to recognize tumor biomarker mesothelin internalize upon binding

**DOI:** 10.1371/journal.pone.0197029

**Published:** 2018-05-08

**Authors:** Allison R. Sirois, Daniela A. Deny, Samantha R. Baierl, Katia S. George, Sarah J. Moore

**Affiliations:** 1 Molecular and Cellular Biology Program, University of Massachusetts, Amherst, Amherst, Massachusetts, United States of America; 2 Picker Engineering Program, Smith College, Northampton, Massachusetts, United States of America; 3 Department of Biochemistry, Smith College, Northampton, Massachusetts, United States of America; 4 Department of Biological Sciences, Smith College, Northampton, Massachusetts, United States of America; Monash University, AUSTRALIA

## Abstract

Mesothelin is a cell surface protein that is overexpressed in numerous cancers, including breast, ovarian, lung, liver, and pancreatic tumors. Aberrant expression of mesothelin has been shown to promote tumor progression and metastasis through interaction with established tumor biomarker CA125. Therefore, molecules that specifically bind to mesothelin have potential therapeutic and diagnostic applications. However, no mesothelin-targeting molecules are currently approved for routine clinical use. While antibodies that target mesothelin are in development, some clinical applications may require a targeting molecule with an alternative protein fold. For example, non-antibody proteins are more suitable for molecular imaging and may facilitate diverse chemical conjugation strategies to create drug delivery complexes. In this work, we engineered variants of the fibronectin type III domain (Fn3) non-antibody protein scaffold to bind to mesothelin with high affinity, using directed evolution and yeast surface display. Lead engineered Fn3 variants were solubly produced and purified from bacterial culture at high yield. Upon specific binding to mesothelin on human cancer cell lines, the engineered Fn3 proteins internalized and co-localized to early endosomes. To our knowledge, this is the first report of non-antibody proteins engineered to bind mesothelin. The results validate that non-antibody proteins can be engineered to bind to tumor biomarker mesothelin, and encourage the continued development of engineered variants for applications such as targeted diagnostics and therapeutics.

## Introduction

In recent years, the focus in cancer drug development has shifted from relatively non-specific cytotoxic agents, to selective, rationally designed, and mechanism-based therapies [1]. Targeted cancer compounds, which are designed to inhibit specific molecular targets or molecular pathways critical for tumor growth and maintenance, are associated with greater efficacy and fewer side effects compared to traditional chemotherapies [2]. Currently, over 75 targeted therapies are approved for clinical use as essential treatments for a variety of malignancies [3,4]. For many cancers, however, targeted therapeutics are not yet available, and it is imperative to develop targeted therapies for patients who do not currently have this treatment option. Furthermore, it has been recognized that a targeted therapy is only effective when a patient’s tumor expresses the molecular target; therefore, companion diagnostics, including molecular imaging agents, are a critical component for developing targeted therapies [5].

Mesothelin (MSLN) is a cell surface protein shown to be overexpressed in many ovarian [6–8], breast [8–10], pancreatic [11–14], liver [15], and lung [16–18] tumors, among others [19], with limited expression in healthy tissues [20]. MSLN has been shown to bind with established cell surface tumor marker MUC16, also known as CA125, leading to increased tumor cell proliferation and metastasis [8,12,21]. Promising results from ongoing efforts in pre-clinical and clinical trials to target MSLN with antibody and antibody derivatives for therapy demonstrate the promise of MSLN-targeting methods [16,17,22]. However, no MSLN-targeting agents have thus far received approval from the US Food and Drug Administration (FDA).

Directed evolution by yeast surface display (YSD) has been used extensively in protein engineering to improve the molecular recognition, biophysical, and catalytic properties of target proteins [23–25]. Directed evolution relies on the generation of mutant libraries followed by identification of mutants with improvements in a desired phenotype by high-throughput screening and selection. The YSD platform offers unique advantages over other directed evolution display formats, including the ability to incorporate post-translational modifications such as glycosylation and disulfide bonds, eukaryotic protein quality control processes, and compatibility with fluorescent-activated cell sorting (FACS) for quantitative discrimination between protein variants. YSD has been used for a wide range of protein classes for a variety of applications, including affinity maturation [26,27], improving thermal stability [28], selecting against cell-based targets [29–31], and epitope mapping [32,33].

While antibodies are widely used for a variety of research and clinical indications, non-antibody protein scaffolds are being developed for research, biotechnology, and medical applications where the inherent properties of antibodies may be limiting. For example, oncological molecular imaging allows clinicians to non-invasively obtain information such as a tumor’s molecular behavior and a patient’s response to treatment [34]. An optimal molecular imaging agent should efficiently localize to the tumor, while rapidly clearing from non-target tissues and organs [35]. Unfortunately, because of their large size and long clearance half-life, antibodies tend to produce undesirable images with high background signals and low contrast [36]. The complex structure of antibodies also poses many challenges when developing chemical strategies for conjugating polymers or drugs for drug delivery applications, such as in the development of antibody-drug conjugates [37].

**Fig 1:**
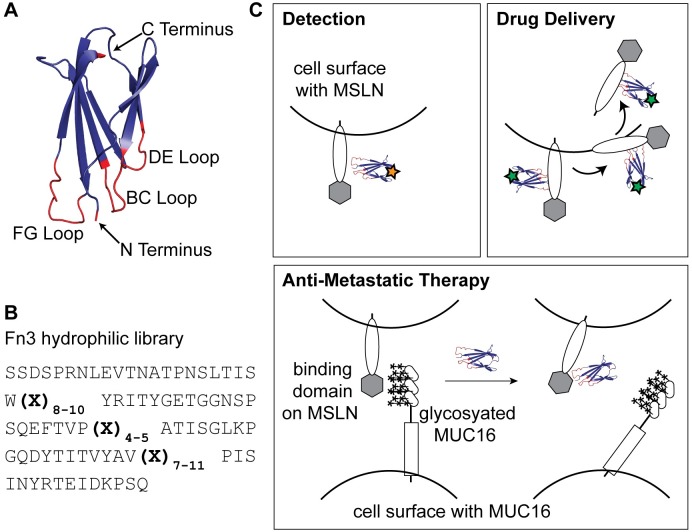
Approach to engineering Fn3 proteins to recognize tumor biomarker MSLN for diagnostic and therapeutic applications. (A) The tenth domain of human fibronectin type III (Fn3) (PDB 1TTG) is a highly stable protein structure with three loops (BC, DE, and FG) broadly tolerant of mutation to confer novel binding properties. Structure was rendered in PyMOL. (B) We employed a previously developed hydrophilic Fn3 yeast surface display library [56] that incorporates a range of loop lengths and biased amino acid composition to mimic the diversity of naturally occurring antibody complementarity-determining regions. (C) Fn3 proteins that bind cell surface protein MSLN have numerous potential clinical applications, such as through diagnostic imaging, internalization for drug delivery, and metastatic reduction by blocking MSLN-MUC16 interactions. Stars represent conjugated imaging or therapeutic molecules.

Efforts to engineer non-antibody, alternative protein scaffolds for molecular recognition have led to binding affinities and specificities once thought to be unique to antibodies [38–43]. Here, we report the engineering of MSLN-binding proteins based on the non-antibody scaffold Fn3, derived from the tenth domain of human fibronectin type III. The hydrophobic core of the immunoglobulin-like fold of Fn3 provides a stable framework structure and high thermostability (T_m_ = 88° C), while the solvent exposed loops of Fn3 are amenable to high diversification (Fig 1A) [44,45]. The Fn3 scaffold has shown great versatility for its ability to be engineered to recognize a variety of targets including ubiquitin [44], epidermal growth factor receptor (EGFR) [46], carcinoembryonic antigen (CEA) [47], human Fc gamma receptors [46], and Abelson kinase Src homology 2 (Abl SH2) domain [48]. Further, engineered Fn3 variants have recently been used for molecular imaging applications, demonstrating the potential of this scaffold as a molecular diagnostic [49,50]. An Fn3 protein that is an antagonist of vascular endothelial growth factor receptor 2 (VEGFR-2) has advanced to Phase II clinical trials, demonstrating the protein scaffold’s promise as a targeted therapeutic [51,52].

While engineered Fn3 clones have high affinity for their targets, some engineered variants have also exhibited oligomeric states or instability [53,54]. Hackel and colleagues demonstrated that an Fn3 YSD library engineered using loop length diversity and recursive mutagenesis could yield highly stable variants that recognized a variety of targets with high affinity [55]. Woldring et al. developed a second generation YSD Fn3 library by incorporating amino acid distributions that recapitulated binding antigens found in nature, which they termed the Gr2 library [56] (Fig 1B). The Gr2 library also incorporated Fn3 framework mutations that increased variant hydrophilicity towards the goal of more desirable in vivo biodistribution for molecular imaging applications [57]. Therefore, the Gr2 Fn3 library is as large in diversity as its parent library, and is designed to be a higher quality protein library. The sequence space sampled is biased toward sequences that are likely to be more successful for identifying high affinity binding variants and for applications in molecular imaging.

Here, we report the engineering of Fn3 variants that bind with high affinity to the MSLN tumor cell surface protein, beginning with the naïve fibronectin YSD Gr2 library. The binding interaction of MSLN and MUC16 is facilitated by non-covalent interactions between the many carbohydrate chains decorating the surface of MUC16 and a minimal binding domain of approximately 64 amino acids on MSLN [58]. Therefore, there is no known native polypeptide sequence that recognizes MSLN, necessitating the use of a naïve protein library as an initial point for our studies. To our knowledge, this is the first non-antibody protein engineered to bind MSLN. The engineered Fn3 variants were expressed and purified at high yields (~10 mg/L) using bacterial culture. Soluble Fn3 variants demonstrated high-affinity binding to tumor cells positive for MSLN expression, and were internalized into tumor cells upon binding. The work described here validates the engineered binding proteins for further development as targeted therapeutics and companion molecular imaging agents (Fig 1C).

## Materials and methods

### Reagents and cell lines

PBSA buffer was composed of phosphate buffered saline (PBS) and 0.1% bovine serum albumin (BSA). *Escherichia coli* (*E*. *coli)* XL1-Blue Supercompetent cells and *E*. *coli* BL21(DE3) cells were purchased from Agilent Technologies and New England Biolabs, respectively. The Gr2 YSD library (generously provided by B. Hackel, University of Minnesota) was grown in selective SD-CAA media containing 20 g/L glucose, 6.7 g/L yeast nitrogen base without amino acids, 5 g/L casamino acids, 7.4 g/L citric acid monohydrate, 10.4 g/L sodium citrate, pH 4.5. SG-CAA media for yeast induction contained 18 g/L galactose, 2 g/L dextrose, 6.7 g/L yeast nitrogen base without amino acids, 5 g/L casamino acids, 5.4 g/L Na_2_HPO_4_, 8.6 g/L NaH_2_PO_4_·H_2_O, pH 6.0. A431/H9 cells (gift of M. Ho, National Cancer Institute, 2016) [59] were cultured in RPMI-1640 (Gibco) supplemented with 10% FBS, 1% penicillin-streptomycin and 700 μg/mL Geneticin selective antibiotic (G418) (Thermo Fisher). KB-3-1 cells (gift of M. Gottesman, National Cancer Institute, 2016) [60], and MCF-7 cells (ATCC #HTB-22, gift of S. Peyton, UMass Amherst, 2017) were cultured in DMEM (Gibco) supplemented with 10% FBS and 1% penicillin-streptomycin.

### Maturation and evolution of mesothelin binders

The naïve Gr2 library (2.8 x 10^9^ diversity), in which EBY100 yeast cells were transformed with the pCT surface display vector encoding for Fn3 variants [56], was sorted and affinity matured generally as previously described [61]. Briefly, the induced library was sorted twice by magnetic bead selection with depletion of non-specific binders using Dynabeads Biotin Binder magnetic beads (Life Technologies). This step served as a negative selection by depleting yeast that displayed Fn3 binders to bare beads or streptavidin. The negative sort was followed by enrichment of specific binding variants by magnetic beads functionalized with biotinylated Fc-tagged recombinant human MSLN (Acro Biosystems #MSN-H826x). The magnetic sorts were followed by a fluorescent-activated cell sorting (FACS) selection for full-length clones using an antibody against the C-terminal c-myc epitope tag (clone 9E10, Life Technologies, 1:50) and a goat anti-mouse phycoerythrin (PE) conjugate (Sigma #P9670, 1:25). Full-length clones were induced and incubated with a chicken anti-c-myc antibody (Gallus Immunotech #ACMYC, 1:330) and the biotinylated Fc-tagged MSLN. To increase the sorting stringency, concentrations of MSLN were decreased over sorting rounds from 300 nM in the first generation sorting to 10 nM by the fourth sort of the second generation library. Cells were washed and incubated with a goat anti-chicken Alexa Fluor 647 (AF647) conjugate (Thermo Fisher #A-21449, 1:250) and either Alexa Fluor 488 (AF488)-conjugated streptavidin (Thermo Fisher #S11223, 1:700) to detect the biotin molecules of the biotinylated Fc-tagged MSLN, or a goat anti-human IgG Fc FITC conjugate (Thermo Fisher #A18830, 1:500) to detect the human Fc domain of the biotinylated Fc-tagged MSLN. Alternating between the two sorting detection methods served to minimize the likelihood of engineering Fn3 variants that bound streptavidin. Cells were washed and double-positive yeast cells were collected on a BD BioSciences FACSAria II. Four iterative rounds of enrichment were performed. Plasmid DNA from the enriched library was recovered using a Zymoprep Yeast Plasmid Miniprep II kit (Zymo Research) following manufacturer’s protocol, transformed into bacteria, and individual clones were sequenced by standard Sanger DNA sequencing methods. Plasmid DNA was subsequently mutated by error-prone PCR of either the entire Fn3 gene or the paratope loops using nucleotide analogues, 8-oxo-2’-deoxyguanosine-5’-triphosphate (8-oxo-dGTP) (TriLink Biotechnologies) and 2’deoxy-p-nucleoside-5’-triphosphate (dPTP) (TriLink Biotechnologies) [62]. All error prone PCR reactions were conducted using primers previously reported [56]. Reaction components and cycling conditions were identical to those previously described [61] with the following exceptions: Standard *Taq* (Mg-free) Reaction Buffer (New England Biolabs) was substituted as the reaction buffer and MgCl_2_ (New England Biolabs, 1.5mM) was added to each reaction. All error prone PCR reactions were conducted as both 10 and 20 cycle reactions to vary the extent of mutagenesis. Mutated plasmid DNA was then amplified and reintroduced into yeast by electroporation with homologous recombination [61].

### Binding affinity measurements of yeast surface displayed variants

Plasmids for Fn3 variants 1.4.1 and 2.4.1, as well as wild type Fn3 (Fn3 WT), were transformed into EBY100 yeast using the Frozen-EZ Yeast Transformation Kit II (Zymo Research) following manufacturer’s protocol. Yeast were grown in SD-CAA media at 30°C and induced with SG-CAA media at 20°C with aeration. Aliquots of 10^6^ yeast cells were simultaneously labeled with 9E10 mouse anti-c-myc antibody (1:50) and a range of concentrations of either biotinylated MSLN-Fc or biotinylated Fc fragment in a total volume of 50 μL PBSA and incubated for 45 minutes with gentle rotation at 23°C. Cells were washed with PBSA and then incubated with a goat anti-mouse PE (1:25) and streptavidin-Alexa Fluor 488 (1:700) for 20 min with gentle rotation on ice in a total volume of 25 μL PBSA, protected from light. Cells were washed with PBSA, pelleted, and resuspended in PBSA for analysis on an EMD Millipore Guava easyCyte flow cytometer. Mean fluorescence intensity for MSLN binding was determined for yeast cells displaying full length protein using InCyte software (EMD Millipore). Data was plotted and fit with a sigmoidal curve using KaleidaGraph software (Synergy). Dissociation constants (K_D_) were determined as the half-maximal value of the sigmoidal fit for three separate experiments for each protein variant, and the mean and standard deviation for the K_D_ values are reported.

### Engineered Fn3 protein production and purification

Engineered Fn3 genes 1.4.1 and 2.4.1 were cloned into the NheI and BamHI sites of a pET24b(+) expression vector modified to include a C-terminal His_6_-KGSGK tag [61] (provided by B. Hackel, University of Minnesota) and expressed in BL21(DE3) *E*. *coli*. Cultures were grown in LB media at 37°C to an optical density at 600 nm of 1.0 before induction with 0.5 mM Isopropyl-β-D-thiogalactopyranoside (IPTG). Fn3 proteins were induced for 3 h at 30°C. Cells were harvested by centrifugation for 15 min at 3,200*g*, and resuspended in lysis buffer (35 mM Na_2_HPO_4_·dibasic, 15 mM Na_2_HPO_4_·monobasic, 500 mM NaCl, 5 mM CHAPS, 25 mM imidazole, 5% glycerol) supplemented with protease inhibitor (cOmplete, Roche). Cells were incubated on ice for 30 min, lysed by repeated freezing and thawing, then centrifuged at 12,000*g* for 10 min and passed through a 0.45-micron filter. Fn3 proteins were purified by nickel affinity chromatography with Ni-NTA agarose resin (Thermo Fisher). Fn3 variant 1.4.1 was further purified by size exclusion chromatography (SEC) on a Superdex 75 10/300 column (GE Healthcare Life Sciences). Fractions of interest were pooled and concentrated with a centrifugal filter unit with a 3 kDa molecular weight cutoff (EMD Millipore). Due to likely nonspecific adsorption onto our SEC column, Fn3 variant 2.4.1 was alternatively further purified by reversed phase high-performance liquid chromatography (HPLC) on a Hypersil ODS C18 column (Thermo Fisher) using a linear gradient of 90% acetonitrile in water containing 0.1% trifluoroacetic acid. Fn3 variant 2.4.1 fractions were pooled, lyophilized, and resuspended. All protein samples were analyzed by SDS-PAGE on a BioRad ChemiDoc MP imaging system using Image Lab 6.0 software (BioRad).

### Alexa Fluor-488 dye conjugation

Pure, folded Fn3 proteins (1 mg/mL) were incubated with AF488 tetrafluorophenyl ester (Thermo Fisher) in a 0.1 M sodium bicarbonate solution, pH 8.3, at an 8:1 dye/protein molar ratio for 1 hr at 23°C with rotation and protected from light. The resulting AF488-labeled 1.4.1 protein was purified by extensive buffer exchange with PBS using a 3 kDa centrifugal filter unit. Again, because of likely adsorption onto the membrane of the centrifugal filter unit, AF488-labeled 2.4.1 protein was purified with an alternative method, using fluorescent dye removal columns (Thermo Fisher #22858), according to manufacturer’s protocol. Concentrations and degree of labeling (DOL) were determined using UV-Vis spectroscopy, measuring dye absorption at 494 nm (ε = 71,000 cm^-1^ M^-1^).

### Binding affinity measurements of soluble Fn3 protein for MSLN-positive tumor cells

A431/H9 and MCF-7 cells were cultured to 80% confluency, as described above, and detached by 0.25% trypsin-EDTA (Gibco). Aliquots of 10^5^ cells were washed and pelleted at 200*g* for 5 min at 4°C. MSLN expression was detected by a mouse anti-MSLN antibody (clone K1, Abcam, 1:50) and a goat anti-mouse PE conjugate (1:25). Cells were incubated with a range of concentrations of AF488-labeled 1.4.1 and 2.4.1 in a total volume of 25 μL PBSA for 1 h at 23°C with rotation and protected from light. Cells were washed and pelleted as above, resuspended with ice cold PBSA, and fluorescence was analyzed using a Guava easyCyte flow cytometer. Mean fluorescence intensities for Fn3 variant binding were determined using InCyte software. Data was plotted and fit with a sigmoidal curve using KaleidaGraph software. Dissociation constants (K_D_) were determined as the half-maximal value of the sigmoidal fit for three separate experiments, and the mean and standard deviation for the K_D_ are reported.

### Imaging flow cytometry

KB-3-1, A431/H9, and MCF-7 cells were cultured and harvested as described above. Aliquots of 2.5 x 10^6^ cells were washed, pelleted, and incubated with AF488-labeled 1.4.1 or 2.4.1 (1 μM) in a total volume of 25 μL for 1 hr at either 23°C or 37°C with rotation and protected from light. Cells were washed with ice cold PBSA and pelleted as above. Cells were fixed with 4% paraformaldehyde (PFA) in PBS for 15 min at 23°C followed by permeabilization with 0.2% Tween 20 in PBS (PBST) for 20 min at 23°C. Cells were washed twice and pelleted then incubated with an AF647-conjugated rabbit anti-EEA1 antibody (Abcam #196186, 1:50) in a total volume of 50 μL PBST for 30 min at 23°C with rotation and protected from light. EEA1 is an early endosomal marker. Cells were washed, pelleted, and resuspended in 100 μL PBSA. Images were acquired on an Amnis ImageStream X Mark II (EMD Millipore) with a 40X magnification. Collected data (5000 images) were analyzed with IDEAS 6.2 software (EMD Millipore). A compensation matrix was created using single color controls acquired with the brightfield laser turned off. Cells were gated for focused cells with the Gradient RMS feature and for single cells with the area and aspect ratio. Co-localization quantification was determined by the Bright Detail Similarity (BDS) metric in IDEAS software.

## Results

### MSLN-binding Fn3 proteins engineered using yeast surface display and directed evolution

**Fig 2:**
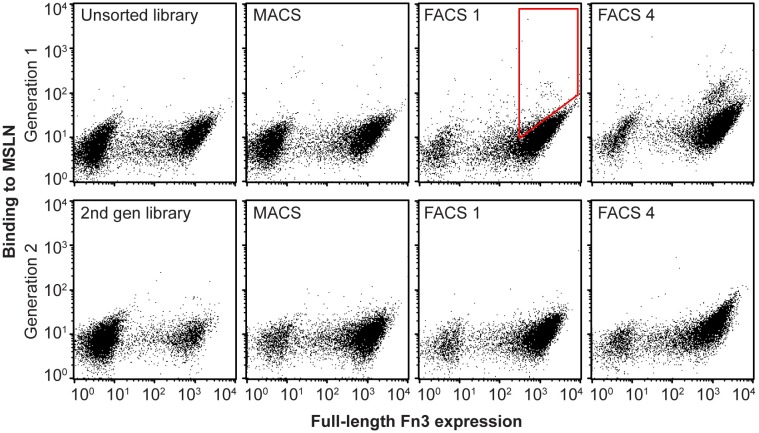
Directed evolution of a naïve yeast surface display library yielded Fn3 variants that bind soluble MSLN. We started with a naïve yeast surface display library with 2.8 x 10^9^ variants of the Fn3 non-antibody scaffold. The library was sorted for full-length protein expression, detected by an antibody to a terminal c-myc epitope tag, and binding to MSLN using MACS and FACS. Red polygon indicates example cell population collected for further enrichment and analysis. Additional diversity was introduced into the enriched library through a single round of mutagenic PCR and sorting of this second generation library resulted in further enrichment for MSLN binding variants. A double-negative population of yeast cells is characteristic of yeast surface display.

Yeast surface display has been previously shown to be a robust method for engineering proteins with improved biophysical, catalytic, and molecular recognition properties [23–25,63]. To engineer MSLN-binding proteins, a naïve Gr2 YSD library of 2.8 x 10^9^ variants was screened. Fn3 expression levels were monitored using an antibody to the terminal c-myc epitope tag. Following two rounds of MACS and a FACS sort for full-length expression to eliminate truncated protein variants, four iterative rounds of dual-color FACS were performed for binding to MSLN normalized by full length protein expression. An enriched population of variants was isolated that demonstrated selective affinity to MSLN, compared to no visible binding in the unsorted naïve library (Fig 2). This enriched population of Fn3 variants was then subjected to a single round of mutagenesis and transformed back into yeast for further enrichment and selection as a second generation library. An enriching population of yeast cells displaying full length protein that bound MSLN was observed throughout rounds of sorting (Fig 2). We note that our initial efforts to engineer Fn3 variants to bind to a small, 64-amino acid domain of MSLN responsible for binding to MUC16 and using an earlier variation of the Fn3 library were unsuccessful, resulting only in variants that bound to the streptavidin secondary reagent and no engineered variants that bound to the MSLN minimal binding domain. To overcome this challenge, we changed our target reagent from this small domain of MSLN to a full-length extracellular domain of MSLN to provide additional surface topology that Fn3 variants could interact with, and alternated sorting detection methods to only use streptavidin as a reagent in some sort rounds, thereby limiting the likelihood of engineering streptavidin binders.

**Fig 3:**
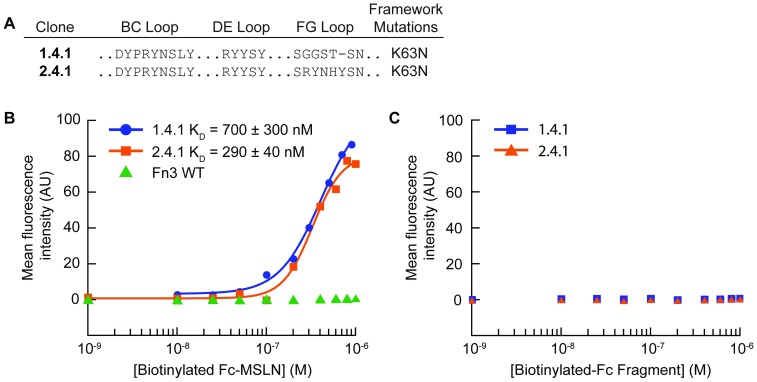
Yeast displayed Fn3 variants 1.4.1 and 2.4.1 bound specifically to tumor biomarker MSLN. (A) Two dominant Fn3 variants, 1.4.1 and 2.4.1, were recovered from a first generation and second generation Fn3 library, respectively. (B) Individual clones and Fn3 WT were displayed on the surface of yeast and incubated with a range of concentrations of soluble MSLN. Experimental triplicate data were collected, and the dissociation constant is reported as the mean and standard deviation of the K_D_ values calculated for each replicate. A representative binding curve is shown for each variant. (C) Individual clones were displayed on the surface of yeast and incubated with a range of concentrations of a biotinylated, Fc fragment. Experimental triplicate data were collected. A representative curve is shown for each variant.

From *E*. *coli* transformed with plasmids obtained from the engineered first and second generation libraries of enriched MSLN-binding Fn3 variants, 30 independent clones from each generation were randomly chosen and sequenced. Following four rounds of dual-color FACS sorting of the first generation library, there were 10 unique sequences. Of those sequences, one unique clone dominated, representing 18 of the 30 clones sequenced, which we refer to as clone 1.4.1, denoting the first generation library, with four rounds of sorting by FACS, clone number one. Following four rounds of sorting of the second generation library, a second unique clone, variant 2.4.1, emerged. Fn3 variants 1.4.1 and 2.4.1 differ only by their FG loop and incorporate a single K63N framework mutation compared to the library wildtype framework sequence (Fig 3A). Plasmids for all unique clones were transformed back into EBY100 yeast and their specific binding to 200 nM MSLN was assessed by flow cytometry. Clones 1.4.1 and 2.4.1 had substantially greater binding to MSLN compared to all other variants, and were subsequently selected for further study.

To measure the binding affinity of these two clones for soluble MSLN extracellular domain, titration binding assays with the Fn3 variants expressed on the surface of yeast were performed (Fig 3B). Clone 1.4.1 exhibited a binding affinity of K_D_ = 700 ± 300 nM, and clone 2.4.1 exhibited a binding affinity of K_D_ = 290 ± 40 nM, while Fn3 WT displayed no specific binding to MSLN. Furthermore, clones 1.4.1 and 2.4.1 exhibited no binding to a biotinylated, Fc fragment alone, demonstrating their specific binding interaction with MSLN (Fig 3C). While further rounds of directed evolution to obtain higher affinity clones will be necessary for eventual clinical translation, we sought to characterize these two variants to learn more about their interaction with tumor cells expressing MSLN, to inform further engineering of Fn3 clones to recognize MSLN.

### Engineered Fn3 proteins were recombinantly produced

**Fig 4:**
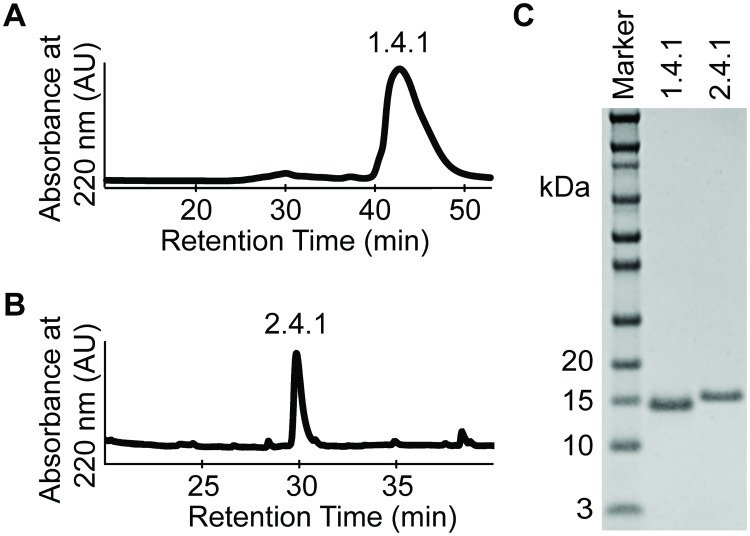
Production and characterization of selected Fn3 variants. Engineered Fn3 clones 1.4.1 and 2.4.1 were expressed in bacteria with a C-terminal hexahistidine tag and a short peptide tag containing GKSK residues for later bioconjugation chemistry. (A) Fn3 protein 1.4.1 was purified by nickel affinity chromatography followed by SEC, demonstrating desired product with retention time of ~ 42 min. (B) Fn3 protein 2.4.1 was purified by nickel affinity chromatography followed by HPLC, demonstrating desired product with retention time of ~30 min. (C) Proteins were purified to high purity > 99% as analyzed by SDS-PAGE. Yields of Fn3 protein production were routinely ~ 10 mg/L.

To further develop and characterize the engineered Fn3 proteins for future diagnostic and therapeutic applications, lead variants were solubly expressed and purified. Fn3 variants 1.4.1 and 2.4.1 were expressed in bacteria with a C-terminal hexahistidine tag and purified by nickel affinity chromatography and either SEC or HPLC. Chromatograms indicated protein elution at the expected retention times for Fn3 variant 1.4.1 on SEC (Fig 4A) and Fn3 variant 2.4.1 on HPLC (Fig 2B). Analysis by SDS-PAGE confirmed high purity > 99% for 1.4.1 and 2.4.1 and (Fig 4C), with routine yields of ~ 10 mg/L.

### Soluble engineered Fn3 variants bound tumor cells expressing MSLN

**Fig 5:**
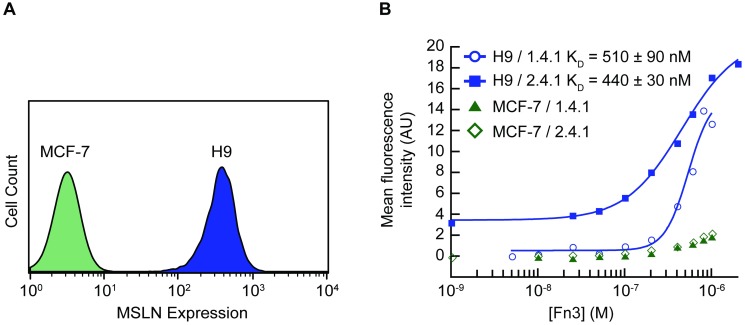
Engineered Fn3 protein variants bound cancer cells expressing MSLN. A431/H9 cells, epidermoid carcinoma cells transfected to express high levels of MSLN, and MCF-7 cells, breast cancer cells lacking surface MSLN, were used in all binding assays. (A) Analysis by flow cytometry confirms MSLN presence on the surface of A431/H9 cells as detected by an anti-MLSN antibody. The MCF-7 cell line does not express MSLN. (B) Fn3 variants 1.4.1 and 2.4.1 were isolated and binding to MSLN was measured using equilibrium binding assays. A431/H9 and MCF-7 cells were incubated with a range of concentrations of soluble fluorescently labeled 1.4.1 or 2.4.1. The assays were performed in experimental triplicate. Data from each replicate were fit to a sigmoidal curve, and a K_D_ value was calculated for each replicate. The K_D_ is reported as the mean +/- standard deviation. A representative binding curve of each clone for both cell lines is shown.

We established a tumor cell binding assay to measure the binding affinities of soluble engineered Fn3 variants for MSLN-expressing cancer cells. The A431/H9 cell line is an A431 (human epidermoid carcinoma) cell line transfected to stably overexpress MSLN on its surface [16]. MCF-7 is a human breast cancer cell line reported not to express MSLN on its surface [7]. Using an anti-MSLN antibody (clone K1, Abcam), high levels of MSLN were confirmed on the surface of A431/H9 cells compared to the MCF-7 cell line expected to be negative for human MSLN (Fig 5A).

Direct equilibrium binding titrations of the engineered 1.4.1 and 2.4.1 Fn3 variants on the A431/H9 and MCF-7 carcinoma cells were performed (Fig 5B). The Fn3 variants were directly conjugated to Alexa Fluor 488 and incubated over a range of concentrations with cells for 1 h at 23°C and analyzed by flow cytometry. Equilibrium binding constant (K_D_) values were obtained by fitting plots of AF488-labeled 1.4.1 and AF488-labeled 2.4.1 concentrations versus the mean fluorescence intensity. Consistent with the yeast surface display binding data, Fn3 variant 1.4.1 bound to A431/H9 cells with a binding affinity of K_D_ = 510 ± 90 nM, while Fn3 variant 2.4.1 bound to A431/H9 cells with a binding affinity of K_D_ = 440 ± 30 nM. Neither Fn3 variant displayed binding to the MSLN-negative MCF-7 cell line, with only expected, non-specific binding observed at the highest concentrations analyzed.

### Fn3 variants co-localized to early endosomes following binding to MSLN

**Fig 6:**
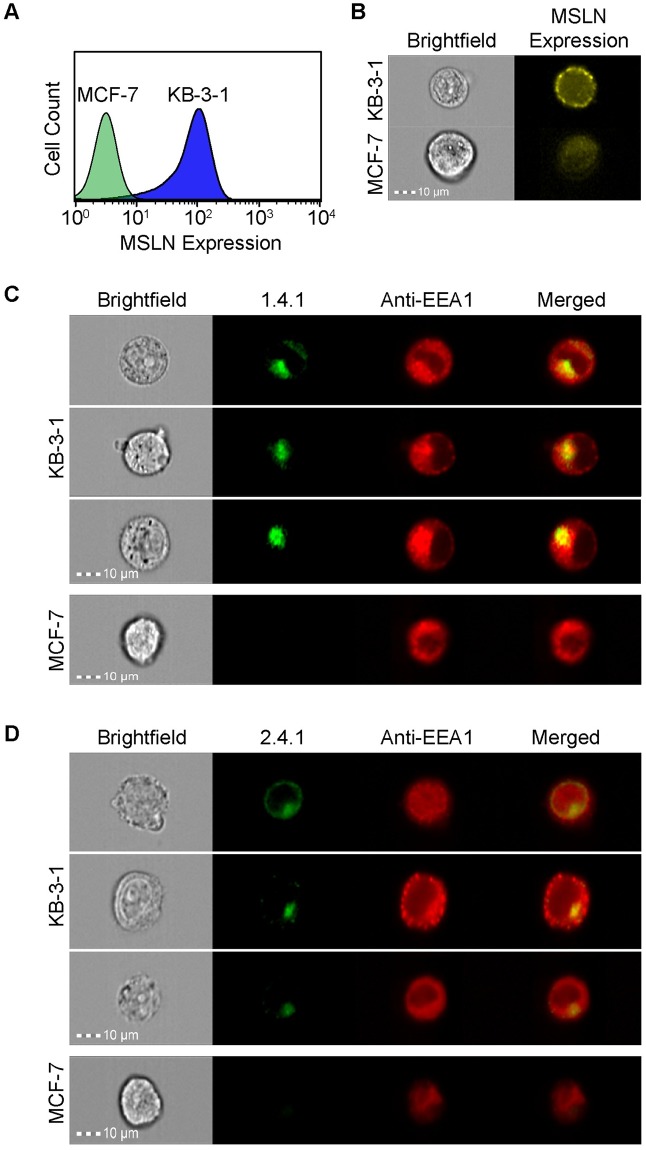
Engineered Fn3 protein variants 1.4.1 and 2.4.1 localized to early endosomes upon binding MSLN. Analysis by (A) flow cytometry and (B) imaging flow cytometry confirms MSLN presence on the surface of KB-3-1 cells compared to the MSLN-negative MCF-7 cells, as detected by an anti-MSLN antibody. (C, D) KB-3-1 cells (*top*) internalize AF488-labeled 1.4.1 (C) and AF488-labeled 2.4.1 (D), while MCF-7 cells show no specific binding or internalization (C *bottom*, D *bottom*). Endosomes are detected by an AF647-conjuated antibody recognizing the EAA1 early endosomal marker. Yellow in the merged images indicate co-localization between AF488-1.4.1 or AF488-2.4.1 anti-MSLN engineered proteins (green) and EEA1 (red). Original magnification 40X. Co-localization is quantified by the Bright Detail Similarity (BDS) metric, with values near 1 indicating co-localization. KB-3-1 BDS = 1.31 and 0.919 for AF488-1.4.1 and AF488-2.4.1, respectively. BDS values are not quantifiable for the negative control cell line, due to insufficient fraction of negative control cell population staining for binding or internalization of engineered protein variants.

Future application of an engineered Fn3 variant for drug delivery to cancer cells could benefit from the internalization of the targeting molecule upon target binding to effectively deliver a conjugated payload into the cells. Mesothelin has been previously reported to efficiently internalize [64–66]. Using imaging flow cytometry, we sought to assess whether engineered Fn3 variants 1.4.1 and 2.4.1 could be internalized into cancer cells following binding to surface MSLN. The KB-3-1 cell line is a human cervical carcinoma cell line reported to express MSLN on its surface [67]. Using an anti-MSLN antibody (clone K1) and a PE-conjugated secondary antibody, MSLN was confirmed on the surface of KB-3-1 cells while no MSLN expression was detected on the surface of the MCF-7 cell line (Fig 6A and 6B).

KB-3-1 cells and MCF-7 cells were incubated with either AF488-labeled 1.4.1 (Fig 6C) or 2.4.1 (Fig 6D) at 37°C and then fixed and permeabilized before incubation with an AF647-conjugated antibody against the early endosomal marker, EEA1. Co-localization of EEA1 antibody and engineered variants was quantified using the Bright Detail Similarity (BDS) metric, which uses a modified Pearson’s correlation coefficient to quantify the degree of similarity between the AF488-labeled 1.4.1 or 2.4.1 image and the AF647-EEA1 endosomal image. Cells with increased AF488-labeled 1.4.1 or 2.4.1 trafficking to early endosomes have higher similarity values as a result of greater co-localization of the two fluorescent channel signals. Imaging flow cytometry data demonstrated that when KB-3-1 cells were incubated with AF488-labeled 1.4.1 (Fig 6C, *top*) or 2.4.1 (Fig 6D, *top*), the AF488-labeled 1.4.1 and 2.4.1 was internalized and co-localized with early endosomes with a BDS = 1.31 and 0.919, respectively, for 5000 cells. Further, efficient binding and subsequent internalization is not observed when AF488-labeled 1.4.1 (Fig 6C, *bottom*) or 2.4.1 (Fig 6D, *bottom*) is incubated with the MSLN-negative MCF-7 cell line. BDS values were not determined for the negative control cell line as this metric requires a substantial population of double positive cells, which was not present for the negative control cell line. In an additional imaging flow cytometry study, MSLN was again confirmed on the surface of KB-3-1 and A431/H9 cells, and AF488-labeled 1.4.1 internalized and co-localized with early endosomes when the experiment was conducted at 23°C (S1 Fig).

## Discussion

Mesothelin has broad potential as a novel tumor target for both diagnosis and therapy, yet no MSLN-targeting molecules are currently FDA approved. Thus, there remains critical need for MSLN-targeting therapeutics and for molecular diagnostics that can identify patients who are most likely to respond to such therapies. In this work, we used directed evolution and a yeast surface display library to engineer Fn3 variants that bind specifically to MSLN, for future application in diagnosis and therapy. Variants 1.4.1 and 2.4.1 demonstrate specific affinity for the MSLN tumor marker present on the surface of tumor cells, and, upon MSLN binding, are internalized and co-localize with early endosomes. Internalization could be valuable for delivery of cytotoxic molecules conjugated to engineered Fn3 variants into tumor cells. The work described here validates our approach for engineering MSLN-binding variants, using yeast surface displayed Fn3 libraries and directed evolution. The results demonstrating specific binding to, and internalization into, a tumor cell line encourage further engineering of higher affinity variants towards clinical applications. To our knowledge, this is the first report of a non-antibody protein engineered to bind MSLN.

In an initial protein engineering strategy, we sought to engineer Fn3 variants that were selected to bind to a 64-amino acid domain of MSLN previously reported to be the minimal domain for binding to MUC16 [58]. It was expected that an Fn3 variant that targeted this binding domain would likely block MSLN and MUC16 interactions, enhancing therapeutic activity of such Fn3 variants. MSLN and MUC16 binding has been reported to enhance tumor cell proliferation and metastasis [8,12,21]. This initial protein engineering strategy did not successfully yield MSLN binding variants, potentially due to insufficient binding topography on the 64-amino acid domain, and, instead, resulted in Fn3 variants that bound the streptavidin secondary reagent. While we had attempted to prevent selecting streptavidin-binding variants by using negative magnetic sorts, this method is not always adequate to influence the selection towards the desired interaction with target protein. Because naïve libraries are not based on pre-existing binding interactions, such libraries display no initial bias toward a specific target molecule or epitope [68]. We were ultimately successful in engineering Fn3 variants that bound to our target protein by using the full-length extracellular domain of MSLN and by avoiding the use of streptavidin in some FACS rounds. Determining the epitopes on MSLN that the engineered variants bind is of interest to further evaluate therapeutic potential of the Fn3 proteins.

Diagnostic molecular imaging is one promising application for non-antibody proteins engineered to bind MSLN positive tumors. While antibodies can be engineered to bind to a variety of targets with high affinities, their large size and slow clearance from circulation can often result in low contrast images [69]. Instead, non-antibody scaffolds have been explored and have demonstrated promising results in preclinical and clinical evaluations [70]. Recently, Fn3 proteins engineered to bind EGFR and EphA2 have been shown to identify tumors expressing their respective molecular target in murine molecular imaging models [50,71,72]. The cystine-knot, or knottin, protein scaffold has also been engineered for tumor targeting applications and has shown promise for molecular imaging in pre-clinical studies targeting tumors and tumor vasculature expressing integrins [73–77]. Likewise, affibodies and DARPins engineered to bind human epidermal growth factor 2 (HER2) or EGFR have been used to image tumor xenografts in mice [78–80]. Recently, a novel Gp2 scaffold has been developed for molecular imaging of EGFR [81]. In each of these studies, the imaging agents were proteins engineered to have picomolar to single-digit nanomolar dissociation constants for their targets, and the importance of this high affinity for tumor targeting applications is further supported by theoretical modeling [82], motivating additional rounds of mutagenesis and directed evolution for our engineered proteins targeting the novel tumor target MSLN.

There is also sustained interest around using engineered proteins as drug delivery agents, such as by conjugating cytotoxic molecules or polymeric systems to proteins that recognize a tumor biomarker [83,84]. Current drug delivery strategies, such as antibody-drug conjugates (ADCs), take advantage of the specificity of antibodies to selectively deliver cytotoxic drugs to antigen-expressing cancer cells [85]. ADCs, including Adcentris^®^ (Seattle Genetics) [86] and Kadcyla^®^ (Genentech)[87], have received FDA approval for targeted treatment of relapsed Hodgkin Lymphoma and Her-2 positive breast cancer, respectively. ADCs are comprised of a targeting antibody, a stable linker with acid labile bonds, and the cytotoxic payload [88]. Upon antigen recognition and binding, the ADC is internalized via receptor-mediated endocytosis and trafficked through endosomal vesicles to the lysosome [89]. The low pH of the lysosome will trigger degradation of the antibody and hydrolysis of the linker, thereby releasing the drug to exert its cytotoxic effect [85]. Dose-limiting toxicities, however, can limit penetration of ADCs into solid tumors, whereas small non-antibody scaffolds may be advantageous by efficiently delivering cytotoxic payloads deep within a tumor while maintaining rapid clearance from circulation [90]. The observed internalization of the MSLN-targeting Fn3 variant is intriguing toward the goal of delivering a payload across the membrane of MSLN-positive tumor cells. Further understanding of the trafficking of engineered proteins that bind MSLN will inform development of anti-MSLN therapeutic strategies.

In summary, we demonstrate that the Fn3 protein scaffold is suitable for engineering targeting molecules for the underdeveloped tumor target MSLN. To our knowledge, this is the first report of a non-antibody protein engineered to bind MSLN. Our data demonstrating specific binding of the engineered variants to tumor cells positive for MSLN, followed by subsequent internalization of the engineered Fn3 proteins, establishes the potential for further development of MSLN-targeting Fn3 proteins for a variety of clinically relevant applications in diagnosis and therapy.

## Supporting information

S1 Fig Engineered Fn3 protein variant 1.4.1 localized to early endosomes in KB-3-1 and A431/H9 cells upon binding MSLN. (A) Analysis by imaging flow cytometry confirms MSLN on the surface of KB-3-1 (*top*) and A431/H9 (*bottom*) cells as detected by an anti-MSLN antibody. (B) KB-3-1 (*top*) and A431/H9 (*bottom*) cells were incubated with AF488-1.4.1 at 23°C for 1 hr. Cells were fixed and permeabilized, then incubated with an AF647-conjugated antibody directed against the early endosomal marker EEA1. Yellow in the merged image indicates co-localization between AF488-1.4.1 anti-MSLN engineered protein (green) and EEA1 (red). Original magnification 40X. Quantification of co-localization for KB-3-1 and A431/H9 as measured by BDS was 0.904 and 0.857, respectively.
(PDF)
