# Supplementary material for: Fn3 proteins engineered to recognize tumor biomarker mesothelin internalize upon binding
Source: PLoS One. 2018 May 8;13(5):e0197029. doi: 10.1371/journal.pone.0197029 (PMC5940182; doi:10.1371/journal.pone.0197029)
Supplement: S1 Fig — (A) Analysis by imaging flow cytometry confirms MSLN on the surface of KB-3-1 (top) and A431/H9 (bottom) cells as detected by an anti-MSLN antibody. (B) KB-3-1 (top) and A431/H9 (bottom) cells were incubated with AF488-1.4.1 at 23°C for 1 hr. Cells were fixed and permeabilized, then incubated with an AF647-conjugated antibody directed against the early endosomal marker EEA1. Yellow in the merged image indicates co-localization between AF488-1.4.1 anti-MSLN engineered protein (green) and EEA1 (red). Original magnification 40X. Quantification of co-localization for KB-3-1 and A431/H9 as measured by BDS was 0.904 and 0.857, respectively. (PDF) [file pone.0197029.s001.pdf]

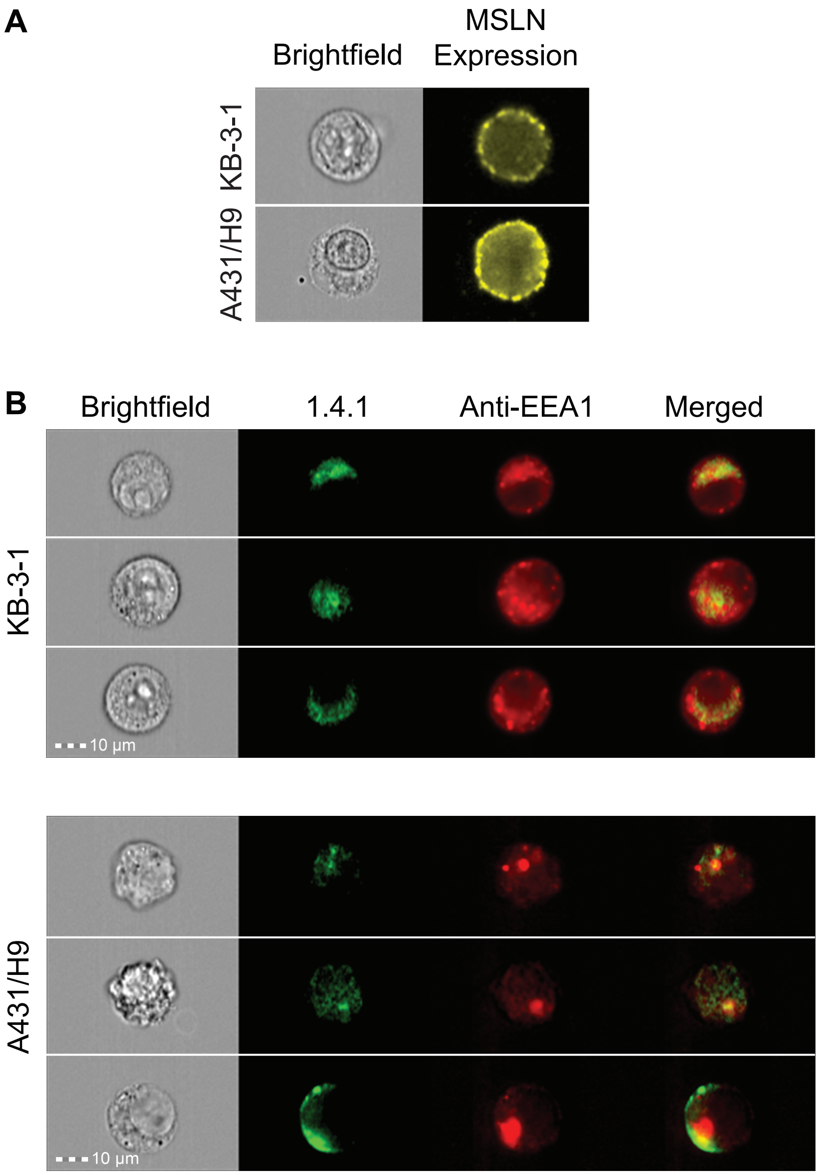

S1 Fig. Engineered Fn3 protein variant 1.4.1 localized to early endosomes in KB-3-1 and A431/H9 cells upon binding MSLN. (A) Analysis by imaging flow cytometry confirms MSLN on the surface of KB-3-1 (*top*) and A431/H9 (*bottom*) cells as detected by an anti-MSLN antibody. (B) KB-3-1 (*top*) and A431/H9 (*bottom*) cells were incubated with AF488-1.4.1 at 23°C for 1 hr. Cells were fixed and permeabilized, then incubated with an AF647-conjugated antibody directed against the early endosomal marker EEA1. Yellow in the merged image indicates co-localization between AF488-1.4.1 anti-MSLN engineered protein (green) and EEA1 (red). Original magnification 40X. Quantification of co-localization for KB-3-1 and A431/H9 as measured by BDS was 0.904 and 0.857, respectively.
